# Haptoglobin treatment prevents cell-free hemoglobin exacerbated mortality in experimental rat sepsis

**DOI:** 10.1186/s40635-021-00387-7

**Published:** 2021-05-03

**Authors:** Christian A. Schaer, Victor Jeger, Thomas Gentinetta, Donat R. Spahn, Florence Vallelian, Alain Rudiger, Dominik J. Schaer

**Affiliations:** 1 Division of Internal Medicine, University and University Hospital Zurich, Raemistrasse 100, 8091 Zurich, Switzerland; 2 Institute of Anesthesiology, University and University Hospital Zurich, Raemistrasse 100, 8091 Zurich, Switzerland; 3 CSL Behring AG, Bern, Switzerland; 4 Department of Medicine, Hospital Limmattal, Urdorferstrasse 100, 8952 Schlieren, Switzerland

Sepsis is a dysregulated host response to infection leading to organ dysfunction, organ failure, and death. Multiple mechanisms promote hemolysis during sepsis, such as complement activation, disseminated intravascular coagulation, hemolytic pathogens, sepsis-induced erythrocyte dysfunction, blood transfusion, and medical procedures with extracorporeal circulation (e.g., renal replacement therapy) [1]. Clinical observations suggested that hemolysis with increased cell-free hemoglobin (CFHb) in plasma correlated with reduced survival in sepsis patients [2–4]. CFHb is a toxin, which may worsen sepsis pathophysiology by nitric oxide depletion, oxidative tissue injury, activation of coagulation and innate immune pathways, and as an iron source for pathogens [5]. The acute phase protein haptoglobin is the archetypical Hb scavenger in plasma and irreversibly neutralizes the toxicity of bound Hb [1].

Here, we performed a prospective, randomized, blinded animal study to provide direct experimental evidence that CFHb exacerbates sepsis mortality and test whether haptoglobin administration could revert this potentially detrimental adverse effect of hemolysis. For this, we used a fluid resuscitated fecal peritonitis model in awake rats that we have characterized in detail earlier (Fig. 1a) [6]. We first validated an Hb-administration protocol in 36 septic rats randomized to saline, CFHb, or Hb–haptoglobin infusion. Three hours after a bolus followed by continuous infusion, the mean total Hb concentrations in plasma were 5.4 μM (SD ± 3.2 μM) in the saline group and 30.4 μM (SD ± 17.3 μM) in the Hb group (Fig. 1b). These data confirmed that our infusion protocol resulted in plasma concentrations within the range of CFHb observed in patients with severe sepsis [2, 4]. Co-administration of human plasma-derived haptoglobin prevented Hb's renal clearance, resulting in higher concentrations than in the CFHb group (54.7 μM ± 63.0 μM). We determined the fractions of CFHb, Hb–haptoglobin complexes, and heme-protein adducts by size-exclusion chromatography. CFHb and heme-protein adducts eluting in the albumin region remained suppressed when haptoglobin was administered concomitantly with CFHb. This confirms that Hb remains stabilized in the Hb–haptoglobin complex for prolonged periods in circulation and that the complex efficiently prevents Hb degradation and heme release from CFHb [7, 8].

**Fig. 1:**
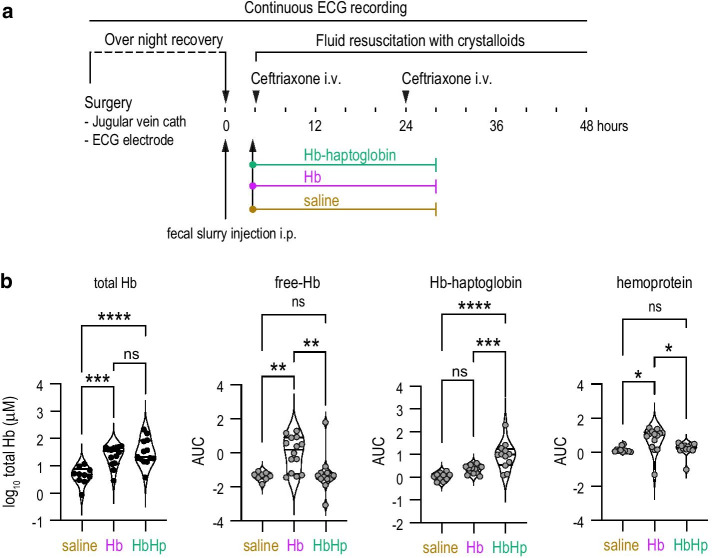
Experimental setup and hemoglobin infusion protocol validation. **a** Setup scheme of the studies: a catheter was placed into the jugular vein and a telemetry ECG electrode was placed subcutaneously the day before the experiment. On the next day, the animals were randomized to a treatment group. Fecal slurry was injected i.p at *T*_0h_ to induce sepsis. 4 h later, the animals were treated with ceftriaxone and a continuous saline infusion was started to provide fluid resuscitation. At the same time (*T*_4h_), we administered a bolus followed by continuous-rate infusion of saline, Hb or Hb–haptoglobin complexes for 24 h (Hb bolus = 16 mg, Hb continuous rate infusion = 15 mg/h, haptoglobin was dosed at an iso-stoichiometric concentration with Hb). **b** Besides the mortality study, we performed a separate study to determine plasma concentration of total Hb by spectrophotometry (left panel black symbols, indicated in heme equivalents), and plasma concentrations of CFHb, Hb–haptoglobin complex and hemoprotein by size-exclusion chromatography (gray symbols). Plasma was collected 3 h after starting the Hb or Hb–haptoglobin infusion (*T*_7h_). *p* values: * < 0.05, ** < 0.01, *** < 0.001, **** < 0.0001

In the main study, we randomized 54 septic Wistar rats to treatment with saline, CFHb, or Hb–haptoglobin. One animal randomized to the saline group had to be excluded from the study, because the intravenous catheter was dislocated during the experiment. In addition, five non-septic animals were infused with CFHb to exclude acute Hb toxicity in healthy animals. After fecal slurry injection, tachycardia developed in all treatment groups consistent with a systemic inflammatory response (i.e., sepsis) (Fig. 2a). The exact timepoint of animal death was determined based on ECG telemetry recordings. The survival data provided evidence for a significantly higher mortality in the group of septic rats infused with CFHb compared to the septic animals infused with only saline (61% versus 12%; *p* = 0.0066). Co-administration of haptoglobin with CFHb improved mortality to 17%, which was not significantly different from the saline infusion group (12%) (Fig. 2b).

**Fig. 2:**
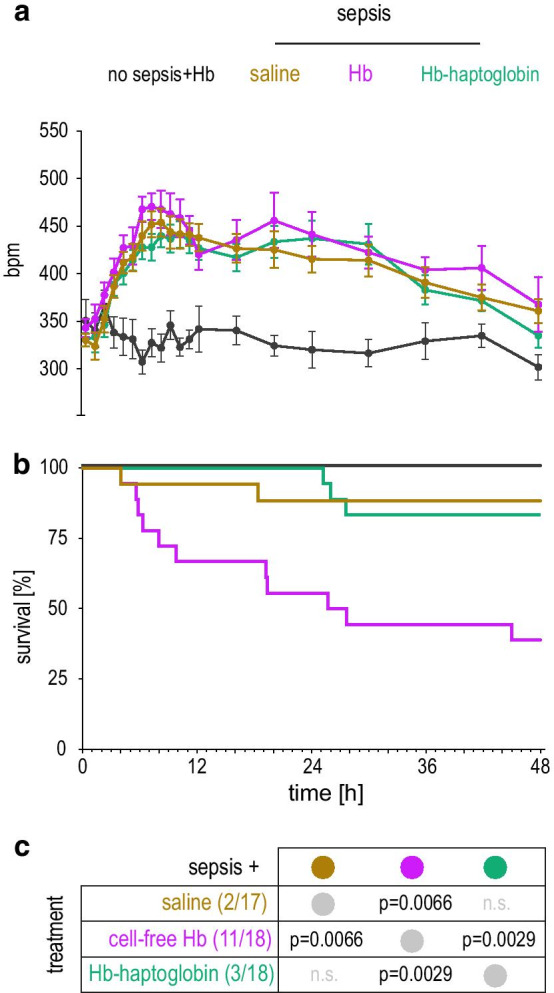
Mortality studies in septic rats. Septic rats were randomized to treatment with a bolus followed by a continuous infusion of saline, Hb, or Hb–haptoglobin over 24 h. All investigators were blinded for the treatment group. An additional group of non-septic rats was infused with Hb to exclude toxicity in healthy animals. **a** Heart rate recordings (mean ± S.E.M) during the 48 h experiments. **b** Kaplan–Meier survival curves. Results of the statistical analysis are provided in the table (**c**). Survival proportions (Kaplan–Meier) were compared using a two-tailed Gehan–Breslow–Wilcoxon test. The familywise significance level of 0.05 was divided by the number of all possible comparisons (*n* = 6), resulting in a Bonferroni-corrected multiple-comparison significance level of 0.00833. [GraphPad Prism software version 8.0 (GraphPad Software, San Diego, CA, USA)]. Numbers of dead animals/total animals per group are given in parentheses

Previous reports demonstrated that blood transfusion-induced hemolysis caused excess mortality in a canine model of *S. aureus* pneumonia [5]. In the same model, administration of a haptoglobin concentrate improved shock, lung injury, and survival, suggesting that Hb-scavenging neutralized the adverse effects of CFHb [5]. With our model, we now provide direct evidence that purified Hb administered to reach clinically relevant plasma concentrations acts as a toxin during hemolysis, mimicking the adverse effect of intrinsic hemolysis. Our data collectively suggest that CFHb is a contributor to adverse sepsis outcomes and may provide a rationale for therapeutic haptoglobin supplementation as a strategy to improve clinical sepsis management.

## Data Availability

Original data are available upon reasonable request from the corresponding author.

## References

[1] Buehler PW, Humar R, Schaer DJ (2020). Haptoglobin therapeutics and compartmentalization of cell-free hemoglobin toxicity. Trends Mol Med.

[2] Janz DR, Bastarache JA, Peterson JF, Sills G, Wickersham N, May AK (2013). Association between cell-free hemoglobin, acetaminophen, and mortality in patients with sepsis: an observational study. Crit Care Med.

[3] Janz DR, Bastarache JA, Sills G, Wickersham N, May AK, Bernard GR (2013). Association between haptoglobin, hemopexin and mortality in adults with sepsis. Crit Care..

[4] Adamzik M, Hamburger T, Petrat F, Peters J, de Groot H, Hartmann M (2012). Free hemoglobin concentration in severe sepsis: methods of measurement and prediction of outcome. Crit Care.

[5] Remy KE, Cortés-Puch I, Solomon SB, Sun J, Pockros BM, Feng J (2018). Haptoglobin improves shock, lung injury, and survival in canine pneumonia. JCI Insight.

[6] Rudiger A, Jeger V, Arrigo M, Schaer CA, Hildenbrand FF, Arras M (2018). Heart rate elevations during early sepsis predict death in fluid-resuscitated rats with fecal peritonitis. Intensive Care Med Exp.

[7] Boretti FS, Buehler PW, Dgnillo F, Kluge K, Glaus T, Butt OI (2009). Sequestration of extracellular hemoglobin within a haptoglobin complex decreases its hypertensive and oxidative effects in dogs and guinea pigs. J Clin Investig..

[8] Deuel JW, Vallelian F, Schaer CA, Puglia M, Buehler PW, Schaer DJ (2015). Different target specificities of haptoglobin and hemopexin define a sequential protection system against vascular hemoglobin toxicity. Free Radic Biol Med.

